# Effects of Ramadan Intermittent Fasting on Gut Hormones and Body Composition in Males with Obesity

**DOI:** 10.3390/ijerph17155600

**Published:** 2020-08-03

**Authors:** Hassane Zouhal, Reza Bagheri, Raoua Triki, Ayoub Saeidi, Alexei Wong, Anthony C. Hackney, Ismail Laher, Katsuhiko Suzuki, Abderraouf Ben Abderrahman

**Affiliations:** 1 Laboratoire Mouvement, Sport, Santé (M2S)-EA 1274, Université Rennes, F-35000 Rennes, France; 2 Department of Exercise Physiology, University of Isfahan, Isfahan 8174673441, Iran; will.fivb@yahoo.com; 3 ISSEP Ksar Said, University of La Manouba, Tunis 2000, Tunisia; raouatriki1995@gmail.com (R.T.); benabderrahmanabderraouf@yahoo.fr (A.B.A.); 4 Department of Physical Education, Damghan Branch, Islamic Azad University, Damghan 3671637849, Iran; saeidi_as68@yahoo.com; 5 Department of Health and Human Performance, Marymount University, Arlington, VA 22207, USA; alexei.wong@gmail.com; 6 Department of Exercise & Sport Science, University of North Carolina, Chapel Hill, NC 27599, USA; thackney@med.unc.edu; 7 Department of Anesthesiology, Pharmacology and Therapeutics, The University of British Columbia, Vancouver, BC V6T 1Z4, Canada; ismail.laher@ubc.ca; 8 Faculty of Sport Sciences, Waseda University, Tokorozawa 359-1192, Japan

**Keywords:** obese, overweight, gut hormones, fasting, body composition

## Abstract

We studied the effects of Ramadan intermittent fasting (RIF) on gut hormones (leptin, glucagon-like peptide-1 (GLP-1), peptide YY (PYY), cholecystokinin (CCK), and ghrelin) in males with obesity. Thirty sedentary males were randomly allocated to either an experimental group (EG, *n* = 15) or a control group (CG, *n* = 15). The EG group completed their Ramadan fasting rituals (30 days), whereas the CG continued with their normal daily habits. Blood samples were collected at four time points: 24 h before the start of Ramadan (T0), on the 15th day of Ramadan (T1), the day after the end of Ramadan (T2) and 21 days after Ramadan (T3). There were significant pre-to-post improvements for leptin (*p* = 0.01, *d* = 1.52), GLP-1 (*p* = 0.022, *d* = 0.75), PYY (*p* = 0.031, *d* = 0.69) and CCK (*p* = 0.027, *d* = 0.81) in the EG, with no interaction effect for ghrelin (*p* = 0.74; *d* = 0.008). No significant changes (*p* > 0.05) occurred in plasma volume variations (ΔPV) after RIF in both EG (−0.03 ± 0.01%) and CG (0.06 ± 0.07%). RIF represents an effective strategy to modify appetite-regulating hormones, leading to improved body composition indices and reduced obesity.

## 1. Introduction

The global rates of obesity continue to escalate, making obesity a health concern worldwide [1]. Among the main factors accelerating the development of obesity is uncontrolled food intake combined with a sedentary lifestyle. Food intake is largely controlled by the hypothalamus, which integrates the neuronal and hormonal signals of eating behaviors, satiety, and caloric intake [2]. Several hormones affecting the brain centers are synthesized and released from peripheral tissues, including intestinal and adipose tissues (adipocytes). Additionally, several peptide hormones present in the gastrointestinal tract also mediate food intake. The main hormones regulating appetite and satiety are leptin, ghrelin, glucagon-like peptide-1 (GLP-1), peptide tyrosine-tyrosine (PYY), and cholecystokinin (CCK) [3,4].

Leptin, a ‘satiety’ hormone, is mainly produced by adipose tissue [5] and the hypothalamus [6] and inhibits hunger. Another key appetite-regulating hormone is ghrelin, a ‘hunger’ hormone produced in the gastrointestinal tract, the brain [7] as well as gastric secretions [8] in order to stimulate the appetite. Furthermore, GLP-1 is mainly produced from intestinal L cells in the gastrointestinal system [9] and the nucleus tractus solitarii of the brain [10]. Analogs of GLP-1 are used for the treatment of type 2 diabetes, and other functions of GLP-1 include reducing appetite and decreasing the quantity and frequency of food consumption, leading to an early feeling of ‘fullness’ [11]. GLP-1 is co-secreted with PYY, another anorexigenic-type peptide, also from endocrine L cells in the distal segment of the small bowel. Finally, CCK is a short-term satiety hormone produced by endocrine I cells in the proximal intestine [2,12]. Obesity is accompanied by altered secretions of leptin, ghrelin, GLP-1, PYY, and CCK, which are in turn associated with accelerated weight gain [13]. 

It is important to alter the abnormal release of these gut peptide hormones in an attempt to ameliorate obesity-related risk factors. Among popular strategies to prevent or improve obesity-related risk factors is intermittent fasting (IF), which entails controlling food intake. IF is defined as a period of eating, followed by a period of fasting. Among the various types of IF is Ramadan IF (RIF), in which millions of Muslims undertake one month of RIF in observance of this religious obligation and abstain from food and liquids during daylight hours from dawn to sunset. Several health benefits have been ascribed to RIF [14,15,16], including decreases in blood pressure [17] and weight in individuals with obesity [14,18]. However, little is known about the alterations of gut hormones’ concentrations in individuals with obesity following RIF. This is important to understand, since RIF could potentially improve the dysregulated secretion of gut peptide hormones in obese individuals and improve their health. To date, there are only three studies evaluating the effects of RIF on gut-released hormones [19,20,21]. Mushtaq et al. found that RIF decreased leptin concentrations in obese and overweight individuals [21], while Haghighi et al. reported that GLP-1 concentrations remained unchanged after RIF [20]. In addition, serum concentrations of ghrelin and PYY were not significantly changed in obese females following RIF [19]. Taken together, studies on RIF report equivocal results. Importantly, there are some concerns with the design of these studies such as the lack of control groups (e.g., individuals with obesity who did not fast), which can compromise the validity of previous findings. 

This study investigated the effects of RIF on gut hormones (leptin, ghrelin, GLP-1, PYY, and CCK) in males with obesity in Tunisia. We hypothesized that RIF positively regulates gut hormone release to control appetite and improve body composition in individuals with obesity in Tunisia. 

## 2. Materials and Methods

### 2.1. Participants

Fifty-three sedentary males with obesity (age range: 20–30 years; body mass index, BMI: ≥30 kg/m^2^) were recruited from University students and staff as well as via social networks. Thirty-four participants (age = 24.2 ± 3.6 years) without chronic diseases or other health concerns consented to participate in this study. The inclusion criteria for this study were: A BMI of 30–40 kg/m^2^, no use of drugs or alcohol, lack of regular exercise for at least 6 months before this study, no history of renal, hepatic, cardiovascular diseases and diabetes, and no injuries or other physical disabilities [22]. The exclusion criteria were diabetes or evidence of clinical depression, cognitive disorders, heart disease, cancer, liver or renal disease, chronic pulmonary disease, uncontrolled hypertension, physical disability, or other contraindications. Women were also excluded from this study because of their menstrual cycles for which they are exempted from fasting during Ramadan. A physician evaluated all criteria using the physical activity readiness questionnaire (PAR-Q) and medical health/history questionnaire. This study was approved by the Ethical Committee on Human Research of the University of Rennes 2, France, and the University of la Manouba, Tunisia (ethic code: Tn, UM2019-73), and was carried out in accordance with the Declaration of Helsinki.

### 2.2. Study Design

This study was conducted during Ramadan (from 5 May 2019 to 3 June 2019) in Tunisia and all the measurements (e.g., anthropometric measurements, questionnaires, blood sampling and analysis) were performed at the Laboratory of Biological Analysis, Principal Military Hospital (Tunis, Tunisia).

**Figure 1:**
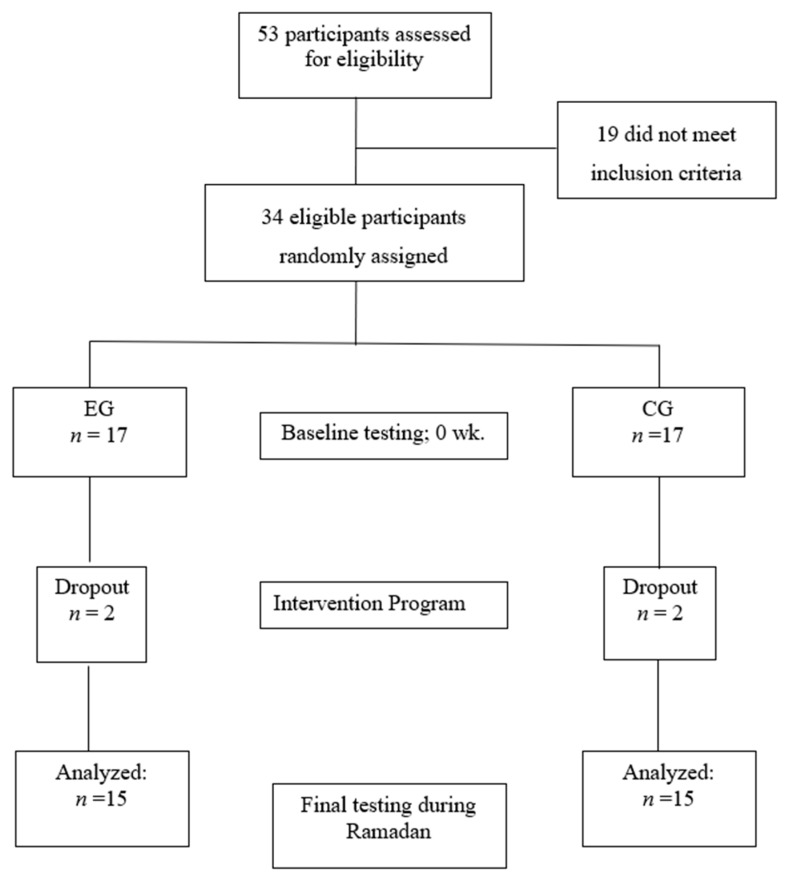
Participant recruitment and allocation to study groups.

**Figure 2:**
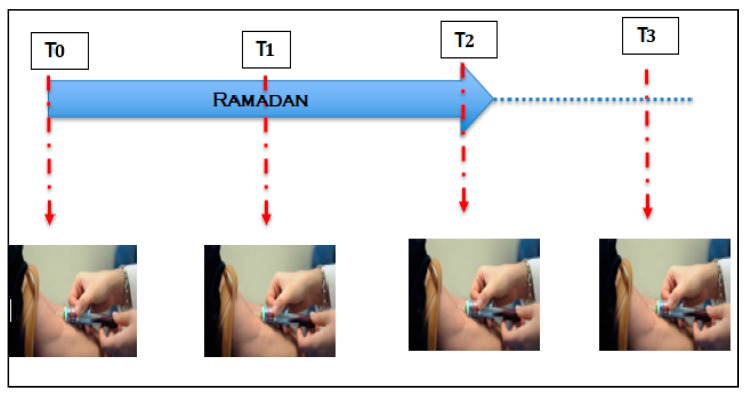
Time points for measurements of anthropometric characteristics and blood sampling at T0 (24 h before the start of Ramadan), T1 (15th day of Ramadan,), T2 (the day after the end of Ramadan) and T3 (21 days after the end of Ramadan).

The fasting duration was 15–16 h per day and in an environment in which the mean temperature was 24 °C. Participants were randomly allocated to either an experimental group (EG, *n* = 15, 24.5 ± 3.8 years) for participants who confirmed that they would fast for the entire month of Ramadan or to a control group (CG, *n* = 15, 23.8 ± 3.7 years) for those who declared that they would not fast during Ramadan) (Figure 1). The EG completed their fasting rituals for the entire month of Ramadan (30 days), whereas the CG continued with their normal daily habits. Prior to any measurements, participants were fully familiarized with all procedures involved in this study. Anthropometric measurements and blood samples were collected at four time points: 24 h before the start of Ramadan (T0), on the 15th day of Ramadan (T1), the day after the end of Ramadan (T2) and 21 days after the end of Ramadan (T3) (Figure 2). All measurements were obtained at the same time of day (within ~1 h). 

### 2.3. Physical Activity Level

Physical activity levels of the participants were classified according to the American College of Sports Medicine Guidelines [23] using the short version of the International Physical Activity Questionnaire (IPAQ) [24]. Physically inactive subjects included those who performed less than 150 min per week of moderate-intensity physical activity and less than 75 min per week of vigorous-intensity physical activity during the last three months, while the physically active subjects met at least one of these criteria [23]. Participants in our study were categorized as being physically inactive.

### 2.4. Dietary Habits Control

All participants were asked to record their normal nutritional habits during the experiment. Participants were asked to complete a two-day 24 h dietary recall, one on a weekday and the other on a weekend day, prior to each blood sampling. For this, participants were asked to recall the foods consumed over the previous 24 h. A 24 h period was defined as the time between when the participants woke up the previous day until the time they woke up the day of the interview [25]. A dietitian informed participants about the recall procedure at the beginning of each interview [25]. The validity and reliability of this method have been previously described [26,27,28]. The number of nutrients consumed was calculated using the method described previously [29]. Subjects in the CG were instructed not to alter their daily dietary habits. Table 1 indicates the nutrients consumed by participants during the study.

### 2.5. Anthropometric Measurements

Body weights were measured on a digital scale (Medtrue Enterprise Co., Ltd., Jiangsu, China) with a precision of 0.1 kg. The height of the participants was measured with a stadiometer (Med. Electronics, Beltsville, MD, USA) with a precision of 0.1 cm. BMI was calculated and recorded as kg/m^2^. Waist circumference was measured using a retractable measuring tape (The perfect measuring company, Toledo, OH, USA) at the superior edge of the iliac crest and around the widest part of the hips; these measurements were used to calculate the waist-to-hip ratio (WHR) of each participant. Body fat percentage (BFP) was measured with a multi-frequency bioelectrical impedance device (Inbody 720, Seoul, South Korea) [30], which had been validated in other studies [31]. However, this method is not as accurate as dual x-ray absorptiometry, which is considered the gold standard for body composition assessment [32]. Fat-free mass (FFM) was calculated by subtracting the fat mass from the body mass. 

### 2.6. Blood Sampling and Analysis

Participants were instructed to comply with the following conditions before each blood sampling: (1) avoid the use of medications and supplements, (2) avoid any strenuous physical activity for at least 72 h before the blood withdrawal, (3) maintain the same diet for 48 h prior to each blood withdrawal, (4) eat the last meal prior to blood sampling before midnight. Blood was collected from the antecubital vein in the morning between 8 and 9 a.m. after an overnight fast. Samples were collected in two pre-cooled 4.9 mL EDTA vacuettes (Klap activator model, Fisher Scientific Inc., Illkirch, France), and whole blood samples were analyzed for a complete blood count using an automated cell counter (Nexcelom Bioscience LLC., Lawrence, KS, USA). Hematocrit (Ht) and hemoglobin concentrations ([Hb]) were then determined. Plasma volume variations (ΔPV) were calculated according to the formula proposed by Dill and Costill (1974):

ΔPV (%) = 100 *×* [([Hb]_a_/[Hb]_b_) *×* [(1 − Ht_b_)/(1 − Ht_a_)] − 100] (1)

‘a’ and ‘b’ refer to fasting state values for Ht and [Hb] before and after fasting, respectively.

One EDTA vacuette was immediately centrifuged at 1500× *g* for 10 min at 4 °C (Heraeus Multifuge X3R, Thermo Scientific, Loughborough, UK). The plasma supernatant was then dispensed into separate 2 mL cryovials and stored at −80 °C until later analysis of total PYY, total GLP-1, CCK and leptin concentrations. From each sample, duplicate 20 μL blood samples were collected into heparinized microhematocrit tubes for determination of hematocrit and a 10 μL sample into a microcuvette for determination of hemoglobin concentrations to enable an estimation of plasma volume changes. To prevent the degradation of acylated ghrelin, a 50 μL solution containing potassium phosphate buffer, p-hydroxymercuribenzoic acid, and sodium hydroxide was added to one 4.9 mL EDTA vacuette, which was then centrifuged at 1500× *g* for 10 min at 4 °C [33]. The plasma supernatant was then dispensed into a storage tube and 100 μL of 1 M hydrochloric acid was added per ml of plasma to preserve acylated ghrelin [34]. 

Commercially available enzyme immunoassays were used to determine plasma concentrations of acylated ghrelin, CCK and leptin concentrations (SPI BIO, Montigny le Bretonneux, France) and total GLP-1, total PYY (Millipore, Watford, UK), and total GLP-1 (Millipore, Watford, UK). To eliminate interassay variation, samples from each participant were analyzed in the same assay batch. The within batch coefficients of variation for the assays were as follows: Acylated ghrelin, 4.3%; total PYY, 5.1%; GLP-1, 4.2%; leptin, 2.9%; and CCK, 1.8%.

### 2.7. Statistical Analysis

A priori power analysis (desired power = 0.80, and alpha error = 0.05) was computed to estimate a statistically significant group by a time-interaction effect based on previous research. The analysis revealed a sample size of *n* = 10.76 per group. To account for potential dropouts, 17 participants were included in each of the two study groups. 

Data are presented as means ± standard deviations (M ± SD). After normality of data distribution was confirmed using the Shapiro–Wilk test, differences within and between groups were calculated using a two-way analysis of variance (ANOVA) for repeated measures. If the group x time interactions were significant, a Newman–Keul’s post-hoc test was calculated. Relationships between parameters were assessed using Pearson’s product-moment correlation coefficient (*r*). Additionally, effect sizes (ES) were determined from ANOVA output by converting partial eta-squared to Cohen’s *d*. Moreover, within-group ES were computed using the following equation: ES = (mean post−mean pre)/SD [35]. In accordance with Hopkins and colleagues’ study, ES were considered trivial (<0.2), small (0.2–0.6), moderate (0.6–1.2), large (1.2–2.0) and very large (2.0–4.0) [36]. The level of significance was set at *p* < 0.05. All statistical analyses were computed using SPSS for Windows, version 16.0 (SPSS Inc., Chicago, IL, USA).

## 3. Results

Four participants (two participants per group) were removed from this study due to non-compliance. The remaining participants reported a 100% adherence to study procedures and conditions. Hence, data are presented herein for the 15 participants per experimental group. No significant between-group differences were noted for any anthropometric and hormone measurements at baseline (T0). Macronutrient values and total energy intake of the EG and CG groups were recorded at T0, T1, T2, and T3 and are described in Table 1. There were no significant differences in macronutrient values and total energy intake between the two groups at each measurement time.

### 3.1. Time-Related Effects

All anthropometric characteristics (Table 2) displayed significant time-related effects (post-test > pre-test). ES magnitudes ranged from small to moderate for all measurements. Significant effects of time were observed for body mass (*p* = 0.007; *d* = 0.484), BMI (*p* = 0.003; *d* = 0.479), BFP (*p* = 0.005; *d* = 0.653), FFM (*p* = 0.002; *d* = 0.690) and WHR (*p* = 0.001; *d* = 0.785). No significant change (*p* > 0.05) of ΔPV was recorded after RIF for both EG (−0.03 ± 0.01%) and CG (0.06 ± 0.07%).

**Figure 3:**
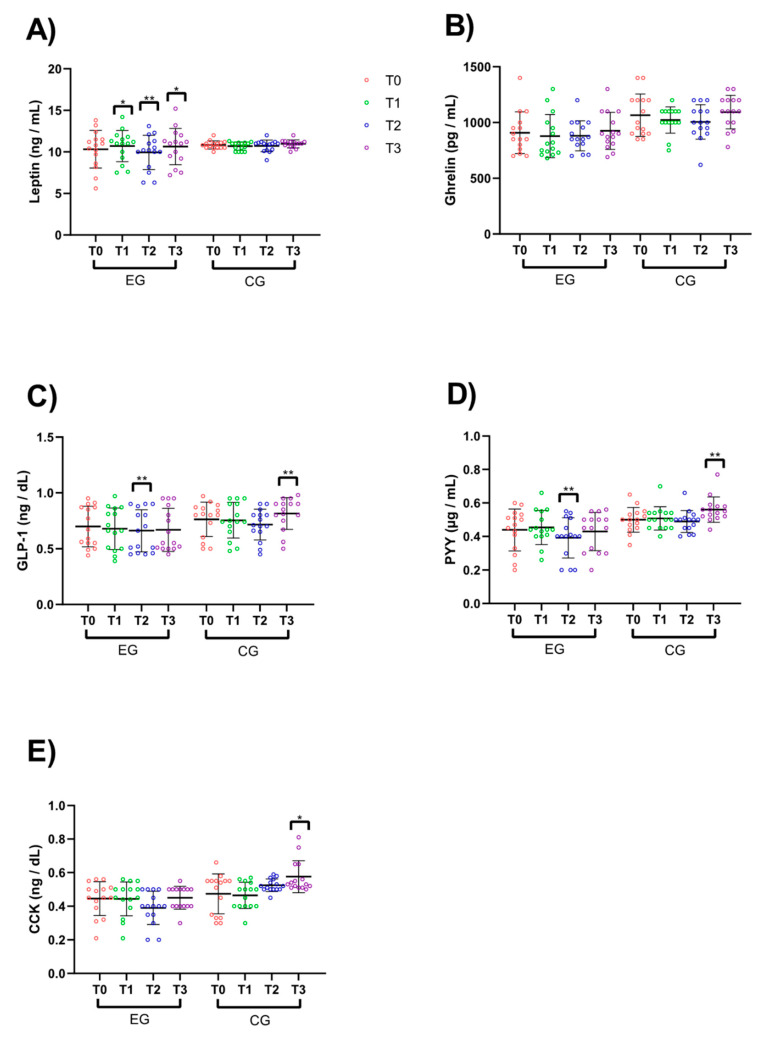
Hormone concentration values. (**A**) Leptin, (**B**) ghrelin, (**C**) glucagon-like peptide-1 (GLP-1), (**D**) peptide YY (PYY), and (**E**) cholecystokinin (CCK). * Significant difference from T0. * *p* < 0.05; ** *p* < 0.01.

There were also significant time-related effects across RIF for all hormone concentrations except for ghrelin. ES magnitudes ranged from trivial to small for all measurements and are shown in Figure 3: leptin (Figure 3A; *p* = 0.004; *d* = 0.15), GLP-1 (Figure 3C; *p* = 0.01; *d* = 0.14), PYY (Figure 3D; *p* = 0.004; *d* = 0.16), and CCK (Figure 3E; *p* = 0.001; *d* = 0.18), with no effect for ghrelin levels (Figure 3B; *p* = 0.08; *d* = 0.09).

### 3.2. Interaction Effects

The group x time interactions for the anthropometric measurements are shown in Table 2. There were significant interactions for body mass (*p* = 0.001; *d* = 0.47), BMI (*p* = 0.001; *d* = 0.48), BFP (*p* = 0.001; *d* = 0.68), FFM (*p* = 0.002; *d* = 0.63) and WHR (*p* = 0.001; *d* = 0.75) (Table 2). Specifically, for EG, post-hoc tests indicated significant pre-to-post declines in body mass (−3.2%, *p* = 0.003; *d* = 1.07), BMI (−3.1%, *p* = 0.007; *d* = 0.75), BFP (−5.8%, *p* = 0.009; *d* = 1.19) and WHR (−6.2%, *p* = 0.006; *d* = 1.12). 

The group x time interactions for hormone concentrations are shown in Table 3. There were significant interactions for leptin (*p* = 0.02; *d* = 0.21), GLP-1 (*p* = 0.024; *d* = 0.32), PYY (*p* = 0.029; *d* = 0.23) and CCK (*p* = 0.001; *d* = 0.21) (Table 3). Specifically, for EG, post-hoc tests revealed significant pre-to-post improvements for leptin (*p* = 0.01; *d* = 1.52), GLP-1 (*p* = 0.022; *d* = 0.75), PYY (*p* = 0.031; *d* = 0.69) and CCK (*p* = 0.027; *d* = 0.81), with no interaction effect for ghrelin (*p* = 0.74; *d* = 0.008).

The interrelationships between percent changes of anthropometric measurements during Ramadan and those of gut hormones in the EG are shown in Table 4. Significant relationships were observed between changes of body mass and BMI and leptin, respectively (*r* = −0.632; *p* = 0.012 and *r* = −0.634; *p* = 0.015); and, between changes of hip circumference and ghrelin (*r* = 0.518; *p* = 0.048).

## 4. Discussion

Our principal finding is that RIF has a beneficial effect on gut hormone levels (leptin, GLP-1, PYY, and CCK) as well as body composition characteristics in obese sedentary men. Our findings suggest that IF could be used as part of an integrated program for combatting obesity in men.

The significant reduction in body mass after RIF is likely due to the reductions in BFP and/or in lean body mass including body water [37]. The loss of body mass we observed can be explained by the reduction in BFP since we recorded a significant reduction in BFP without any significant changes in FFM or PV. The reduction in body weight and visceral adiposity after RIF could be attributed to the metabolic transfer to ketogenesis and the oxidation of fatty acids as an alternate source of energy after the depletion of carbohydrate reserves in the body in response to the prolonged fasting [38,39,40,41]. RIF can also increase liver glycogen mobilization, increase gluconeogenesis, and ultimately increase free fatty acids (FFAs), and in turn, lead to decreases in BFP [42]. Other studies have attributed this decrease to significant dehydration in individuals with obesity during RIF, since such individuals may have more body water due to larger total glycogen stores [43,44]. Thus, RIF also appears to increase resting metabolic rate (RMR), which could lead to a possible decrease in BFP [45]. These changes in body weight and BFP could consequently result in the BMI and WHR changes observed following RIF.

Improvements in gut hormone concentration were observed after RIF. Our findings are in agreement with those of Çaklili et al. who also reported that leptin concentrations increased following RIF, suggesting a role for leptin to satiety in RIF [46]. In contrast, leptin concentrations were decreased after 8 weeks of alternate-day fasting during weight loss in obese participants, which was interpreted as the brain becoming less sensitive to meal-generated satiety signals, leading to decreased leptin concentrations [45]. Consequently, more calories would need to be consumed before a sufficient signal is generated to inhibit food intake. Accordingly, decreases in leptin concentrations following weight loss could have consequences of increased appetite, calorie intake, and weight regain. 

Leptin concentrations are thought to reflect the state of nutrition and energy reserve [47], but there is also a diurnal rhythm of leptin secretion linked to meal patterns. Hence, the shifting mealtime during Ramadan could cause a comparable shift in the rhythm of leptin secretion. Thus, lunchtime is moved forward by ~8 h per day during RIF, which can likely alter the circadian pattern of leptin responses [48]. 

We found that plasma ghrelin concentrations were not significantly increased by RIF, confirming observations by others that ghrelin concentrations were not significantly changed following RIF in obese females [19]. Ghrelin concentrations are primarily regulated by food intake. Hence, ghrelin levels increase during fasting (in line with increased hunger) and are generally lower in individuals with higher body weights compared with lean individuals, suggesting a role for ghrelin in the regulation of body weight [49]. 

We report that GLP-1 concentrations decreased after RIF. Contrary to our results, other studies suggested insignificant changes in GLP-1 concentrations after RIF in females and males, suggesting that the hypothalamus most likely adapts to hunger-related to intentional fasting during Ramadan [19,46]. Furthermore, a non-significant decrease in GLP-1 occurs after RIF in obese females [19]. GLP-1 stimulates insulin release from the pancreas, increases pancreatic beta cell volume, and mitigates glucagon release. GLP-1 also increases the feeling of fullness during and between meals by acting on appetite centers in the brain and by slowing the emptying of the stomach [50]. We showed that RIF negatively affects GLP-1. It has been proposed that even a small increase in GLP-1 release after a meal can increase the risk of obesity [50]. We speculate that the positive correlation between GLP-1 levels and BFP we observed may likely be due to a decreased appetite following RIF. 

PYY is secreted into the blood by cells of the ileum and the colon following stimulation by nutrients from ingested food [50]. Our study shows that PYY concentrations decreased significantly following RIF. PYY is released after food intake and works by binding to receptors in the brain, which in turn decreases appetite and creates a sense of fullness after eating. The amount of PYY released into the blood depends on the number of calories eaten, with higher calorie content foods causing a greater release of PYY. Thus, decreasing food intake during the day with RIF could lower PYY concentrations. The secretion of PYY can also be stimulated by digestive juices (such as bile) and the gastrointestinal hormone CCK, which may be involved in appetite regulation by increasing the sensation of fullness in the short term, such as during a meal rather than between meals. We showed that CCK concentrations decreased significantly following RIF, suggesting that not consuming food throughout the day during RIF affects these key hormones that are responsible for food intake and appetite control. 

### Study Limitations

Both GLP-1 and PYY are released post-prandially, with plasma levels peaking 15–20 min after a meal [20]. As only fasting levels of these hormones were measured, our study may have failed to measure the impact of peak levels in the EG. Similarly, ghrelin is suppressed by feeding and an augmented suppression may have been missed in the EG due to our assessment protocol. Furthermore, we evaluated obese male participants and hence cannot generalize our findings to other populations. 

BFP was measured using bioelectrical impedance, a commonly used technique, which determines BFP based on methods developed in normal-weight subjects but this procedure can underestimate BFP in the obese participants in our study [51,52]. Bioelectrical impedance measures conductance, which can be affected by fluid restriction [53]. Therefore, the use of this method could have masked an even greater decrease in BFP change in the EG.

Since we did not collect data beyond 21 days after the end of RIF, it may be premature to conclude that RIF is a suitable strategy for the long-term management of obesity. Nonetheless, our findings support fasting as a behavioral practice that has some degree of utility in the management of obesity. Future research needs to pursue this line of reasoning to determine practicality of the procedure in such applications.

## 5. Conclusions

Obesity is accompanied by increases in adiposity and changes in appetite-regulating hormones. Altering the abnormal release of these hormones may be useful in managing obesity. Ramadan IF improved body composition indices (BFP, WHR, and BMI) in healthy obese males and produced changes in plasma levels of leptin (increased), GLP-1 (decreased), PYY (decreased) and CCK (decreased), with no changes in plasma ghrelin levels. Changes in hormone levels persisted for 3 weeks after the end of Ramadan. Our findings suggest that IF could be used as part of an integrated program for combatting obesity in men. 

## Figures and Tables

**Table 1:** Macronutrient values (means ± SD) of the experimental group (EG) and control group (CG) measured 24 h before the start of Ramadan (T0), on the 15th day of Ramadan (T1), the day after the end of Ramadan (T2) and 21 days after the end of Ramadan (T3).

Macronutrients	Group	T0	T1	T2	T3
Protein (% kcal)	EG	18 ± 11	19 ± 7	18 ± 5	19 ± 10
	CG	17 ± 3	20 ± 5	18 ± 7	19 ± 5
Carbohydrate (% kcal)	EG	47 ± 8	46 ± 8	46 ± 7	47 ± 10
	CG	46 ± 8	45 ± 8	47 ± 7	47 ± 6
Fat (% kcal)	EG	35 ± 4	35 ± 5	36 ± 11	36 ± 5
	CG	37 ± 4	35 ± 4	35 ± 7	34 ± 4
Energy (kcal/day)	EG	2355 ± 266	2475 ± 354	2410 ± 295	2370 ± 310
	CG	2425 ± 296	2510 ± 267	2445 ± 315	2410 ± 355

Data are expressed as means ± SD.

**Table 2:** Anthropometric and body composition characteristics (means ± SD) of the experimental group (EG) and control group (CG) measured 24 h before the start of Ramadan (T0), on the 15th day of Ramadan (T1), the day after the end of Ramadan (T2) and 21 days after the end of Ramadan (T3).

Variables	Group	Phases				*p*-Values (ES)		
		T0	T1	T2	T3	Time	Group	Group × Time
**Body mass (kg)**	EG	97.8 ± 4.5	97.3 ± 4.4	94.5 ***	95.5 ± 4.9 **	0.007 (0.48)	0.014 (0.19)	0.001 (0.47)
	CG	101.4 ± 6.7	101.2 ± 6.79	101.1 ± 7.1	101.2 ± 7.1			
**BMI (kg/m^2^)**	EG	33.3 ± 1.3	33.2 ± 1.2	32.3 ± 1.2 ***	32.6 ± 1.34 ***	0.003 (0.47)	0.039 (0.02)	0.001 (0.48)
	CG	33.5 ± 2.7	33.5 ± 2.6	33.4 ± 2.7	33.5 ± 2.7			
**Body fat percentage (%)**	EG	35.2 ± 1.5	35.2 ± 1.5	33.1 ± 1.6 ***	33.8 ± 1.9 **	0.005 (0.65)	0.09 (0.001)	0.001 (0.68)
	CG	34.2 ± 1.9	34.2 ± 1.88	34.2 ± 1.8	34.3 ± 1.9			
**FFM (kg)**	EG	63.4 ± 3.0	63.1 ±3.2	63.4 ± 3.4 **	63.2 ± 3.7 **	0.002 (0.69)	0.003 (0.75)	0.002 (0.63)
	CG	67.1 ± 4.9	66.9 ± 5.0	66.9 ± 4.9	66.9 ± 4.8			
**WHR (cm^2^)**	EG	0.97 ± 0.01	0.96 ± 0.05	0.91 ± 0.04	0.87 ± 0.04	0.001 (0.78)	0.001 (0.86)	0.001 (0.75)
	CG	0.98 ± 0.04	0.99 ± 0.02	1.00 ± 0.02	1.03 ± 0.02			

Data are expressed as means ± SD. BMI: body mass index; FFM: fat-free mass; WHR: waist-to-hip ratio. ** *p* < 0.01; *** *p* < 0.001; ES: Effect size.

**Table 3:** Gut hormone concentrations (means ± SD) of the experimental group (EG) and control group (CG) measured 24 h before the start of Ramadan (T0), on the 15th day of Ramadan (T1), the day after the end of Ramadan (T2) and 21 days after the end of Ramadan (T3).

Measures	Group	Time of Measurement				*p*-Values (ES)		
		T0	T1	T2	T3	Time	Group	Group × Time
**Leptin (ng/mL)**	**EG**	10.32 ± 2.26	10.70 ± 1.87 *	9.93 ± 2.05 **	10.64 ± 2.18 *	0.004 (0.15)	0.45 (0.02)	0.02 (0.11)
	**CG**	10.84 ± 0.48	10.68 ± 0.48	10.72 ± 0.70	10.96 ± 0.47			
**Ghrelin (pg/mL)**	**EG**	908.66 ± 187.70	877.67 ± 193.71	880.33 ± 135.20	925.33 ± 166.08	0.08 (0.09)	0.06 (0.24)	0.74 (0.008)
	**CG**	1066.00 ± 190.06	1023.00 ± 117.81	1006.00 ± 155.04	1093.00 ± 149.79			
**GLP-1 (ng/dL)**	**EG**	0.69 ± 0.18	0.67 ± 0.18	0.66 ± 0.19 **	0.67 ± 0.19	0.01 (0.14)	0.016 (0.07)	0.02 (0.12)
	**CG**	0.76 ± 0.15	0.75 ± 0.16	0.71 ± 0.14	0.81 ± 0.14 **			
**PYY (µg/mL)**	**EG**	0.43 ± 0.12	0.45 ± 0.10	0.39 ± 0.12 **	0.43 ± 0.11	0.004 (0.16)	0.01 (0.21)	0.02 (0.12)
	**CG**	0.49 ± 0.06	0.50 ± 0.07	0.48 ± 0.06	0.56 ± 0.070 **			
**CCK (ng/dL)**	**EG**	0.44 ± 0.10	0.44 ± 0.10	0.39 ± 0.09 **	0.45 ± 0.06 **	0.001 (0.18)	0.007 (0.23)	0.001 (0.21)
	**CG**	0.47 ± 0.12	0.46 ± 0.70	0.52 ± 0.30	0.57 ± 0.95 **			

GLP-1: glucagon-like peptide-1; PYY: peptide YY; CCK: cholecystokinin; * significant differences from T0. * *p* < 0.05; ** *p* < 0.01; ES: Effect size.

**Table 4:** Relationships between % changes of anthropometric measurements and gut hormones in experimental group (*n* = 15).

Variables	Δ Body Mass (%)	Δ BMI (%)	Δ BFP (%)	Δ Waist (%)	Δ Hip (%)	Δ WHR (%)
**Δ Leptin (%)**	*r* = −0.631 * p = 0.012 *	*r* = −0.643 * p = 0.015 *	*r* = −0.23 *p* = 0.408	*r* = −0.126 *p* = 0.654	*r* = 0.0121 *p* = 0.966	*r* = −0.106 *p* = 0.705
**Δ Ghrelin (%)**	*r* = −0.072 *p* = 0.798	*r* = −0.0721 *p* = 0.798	*r* = 0.148 *p* = 0.598	*r* = 0.12 *p* = 0.670	*r* = 0.518 * p = 0.048	*r* = −0.443 *p* = 0.098
**Δ GLP-1 (%)**	*r* = −0.132 *p* = 0.637	*r* = −0.132 *p* = 0.637	*r* = −0.191 *p* = 0.495	*r* = −0.395 *p* = 0.145	*r* = −0.202 *p* = 0.470	*r* = −0.033 *p* = 0.906
**Δ PYY (%)**	*r* = 0.427 *p* = 0.112	*r* = 0.427 *p* = 0.112	*r* = 0.024 *p* = 0.932	*r* = −0.059 *p* = 0.835	*r* = −0.43 *p* = 0.109	*r* = 0.404 *p* = 0.134
**Δ CCK (%)**	*r* = 0.091 *p* = 0.744	*r* = 0.091 *p* = 0.744	*r* = −0.127 *p* = 0.652	*r* = −0.0894 *p* = 0.752	*r* = −0.134 *p* = 0.634	*r* = 0.0856 *p* = 0.761

GLP-1: glucagon-like peptide-1; PYY: peptide YY; CCK: cholecystokinin; * significant differences. * *p* < 0.05.
